# Reduced Baseline Airway Caliber Relates to Larger Airway Sensitivity to Rostral Fluid Shift in Asthma

**DOI:** 10.3389/fphys.2017.01012

**Published:** 2017-12-12

**Authors:** Swati A. Bhatawadekar, Gabriel Keller, Cristina O. Francisco, Mark D. Inman, Jeffrey J. Fredberg, Susan M. Tarlo, Mathew Stanbrook, Owen D. Lyons, Azadeh Yadollahi

**Affiliations:** ^1^Toronto Rehabilitation Institute, University Health Network, Toronto, ON, Canada; ^2^Faculdade de Medicina, Universidade Federal do Rio de Janeiro, Rio de Janeiro, Brazil; ^3^Faculty of Medicine (Respirology), McMaster University, Hamilton, ON, Canada; ^4^Department of Environmental Health, Harvard T. H. Chan School of Public Health, Boston, MA, United States; ^5^Department of Medicine and Dalla Lana School of Public Health, University of Toronto, ON, Canada; ^6^Toronto Western Hospital, University Health Network, Toronto, ON, Canada; ^7^Department of Medicine (Respirology), University of Toronto, Toronto, ON, Canada; ^8^Institute of Biomaterials and Biomedical Engineering, University of Toronto, Toronto, ON, Canada

**Keywords:** nocturnal asthma, supine position, respiratory resistance, lower airway narrowing, thoracic fluid

## Abstract

**Background:** We have previously shown that when asthmatics go supine, fluid shifts out of the legs, accumulates in the thorax, and exacerbates lower airway narrowing. In the retrospective analysis of our previous work presented here, we test the hypothesis that the sensitivity of this process relates inversely to baseline caliber of the lower airways.

**Methods:** Eighteen healthy (six women) and sixteen asthmatic subjects (nine women) sat for 30 min, and then lay supine for 30 min. While supine, lower body positive pressure (LBPP, 40 mm Hg) was applied to displace fluid from the legs similar in amount to the overnight fluid shift. Respiratory resistance and reactance at 5 Hz (R5 and X5) and leg and thoracic fluid volumes (LFV and TFV) were measured at the beginning and end of the supine period.

**Results:** With LBPP, healthy, and asthmatic subjects had similar changes in the LFV and TFV (*p* = 0.3 and 0.1, respectively). Sensitivity to fluid shift, defined by ΔR5/ΔTFV, was larger in the asthmatics than in the healthy subjects (*p* = 0.0001), and correlated with baseline R5 in the supine position in the asthmatics (*p* = 0.7, *p* = 0.003). No such association was observed in the healthy subjects (*p* = 0.6). In the asthmatics, women showed a greater reduction in X5 than men with LBPP (*p* = 0.009).

**Conclusions:** Smaller baseline airway caliber, as assessed by larger R5, was associated with increased sensitivity to fluid shift in the supine position. We conclude that asthmatics with narrower small airways such as obese asthma patients, women with asthma and those with severe asthma may be more sensitive to the effects fluid shift while supine as during sleep.

## Introduction

In asthma, inflammatory processes or vascular engorgement can cause excess fluid in the airway interstitial and luminal spaces (Yager et al., 1991, 1995). This excess fluid has been recognized as a key contributor to the pathogenesis of asthma. For example, both human and animal studies demonstrate that airway wall edema due to vascular engorgement enhances airway response to contractile agonist (Rolla et al., 1986; Brown et al., 1995a; Pellegrino et al., 2003). This excess fluid can increase airway wall thickness and reduce luminal cross section area (Yager et al., 1995). It has been hypothesized that increased airway wall thickness amplifies airway hyperresponsiveness by narrowing airway lumen or decoupling the airway wall from the surrounding parenchyma and reducing parenchymal tethering forces on the airway wall (Moreno et al., 1986). Moreover, the excess fluid can enter the airway lumen, and increase airway-liquid interface surface tension causing further airway narrowing and possibly closure of the small airways (Yager et al., 1991, 1995; Brown et al., 1995a; Pellegrino et al., 2003).

In humans, changes in the body posture result in significant changes in body fluid distribution. For example, while upright, intravascular fluid leaks into the interstitial space of the legs due to gravitational and Starling forces, and causes an increase in the interstitial and total leg fluid volume (Thompson et al., 1928; Waterfield, 1931; Youmans et al., 1934; Fawcett and Wynn, 1960). On changing to the supine position as during sleep, the fluid is reabsorbed rapidly from the interstitial space to the intravascular space of the legs (Thompson et al., 1928; Fawcett and Wynn, 1960), and redistributed to the upper body, including to the thorax and neck. We refer here to this process as a rostral fluid shift (Bhatawadekar et al., 2017). Evidence suggests that in asthma, nocturnal rostral fluid shift to the thorax during sleep may play a role in the pathogenesis of overnight airway narrowing in asthma. Previously, Desjardin et al. observed that in asthmatic subjects with nocturnal worsening, pulmonary capillary blood volume increased overnight during sleep in association with an increase in the lower airway narrowing (Desjardin et al., 1995). However, this did not occur in healthy subjects and in asthmatic subjects without nocturnal worsening. In our recent study (Bhatawadekar et al., 2017), we used lower body positive pressure (LBPP) to simulate overnight rostral fluid shift from the legs to the upper body as occurs during sleep (Redolfi et al., 2009). In that study, we found that thoracic fluid volume increased significantly with LBPP, and for similar amounts of fluid accumulation in the thorax, asthmatic subjects experienced a greater increase in the resistance and stiffness of the respiratory system than do healthy subjects.

Since there is a strong inverse relationship between airway diameter and airway resistance (West, 2008), it may be expected that these differences in airway response to rostral fluid shifts between healthy and asthmatic subjects may be related to the differences in baseline airway caliber. Indeed, in our recent study (Bhatawadekar et al., 2017), as well as in other studies (Duggan et al., 2004; Cavalcanti et al., 2006), asthmatic subjects had greater respiratory resistance and stiffness than did healthy subjects at baseline in both upright and supine positions, thus implying differences in the baseline airway caliber. Therefore, it may be concluded that for similar amount of fluid accumulation in the lower airways walls, airways with a smaller lumen will have more narrowing and consequently more pronounced increase in their resistance compared to airways with a larger lumen.

Here, we present a retrospective analysis of our original study (Bhatawadekar et al., 2017) to determine the effects of baseline airway caliber on airway response to rostral fluid shift in asthma. We hypothesize that smaller baseline lower airway caliber, as assessed by higher respiratory system resistance, amplifies the effects of fluid shift on lower airway narrowing in asthma.

## Methods

### Subjects

Subjects were between 30 and 75 years of age and never-smokers or ex-smokers with <10 pack-years of smoking history. Healthy subjects had no history of asthma or any other respiratory condition. Asthmatic subjects with objective evidence of asthma were enrolled from the Respirology clinics at University Health Network and St. Michael's Hospitals, Toronto. They had no history of hypertension, heart failure, any cardiovascular, renal, or neurological condition or were not on medications for these conditions or medications that may affect fluid retention (e.g., diuretics). All female subjects underwent the study measurements outside their menstrual period. All subjects gave written, informed consent before participating. The study protocol was approved by institutional ethics boards.

### Inducing rostral fluid shift

While in the supine position, we applied LBPP (LBPP, 40 mm Hg) to induce rostral fluid shift similar in amount to the fluid shift that occurs during overnight sleep (Bhatawadekar et al., 2017). LBPP is a well-established technique for displacing fluid from the lower extremities to the upper body (Bivins et al., 1982). With subjects lying supine, deflated medical antishock trousers (MAST III–AT; David Clark, Inc., Worcester, MA) were wrapped around subjects' legs from the hips to the ankles, and inflated to 40 mmHg. This pressure was sustained for 20 min. The trousers remained deflated in the control study arm.

### Measuring legs and thoracic fluid volumes

The legs and thoracic fluid volumes (LFV and TFV, respectively) were measured with the bioelectrical impedance technique (Biopac Systems EBI100C; Yadollahi et al., 2015; Bhatawadekar et al., 2017). This is based on Ohm's law that resistance of a segment is inversely related to the amount of fluid in the segment. A small amplitude current (400 μA) was injected in the right leg and thorax (frequencies, 25 and 100 kHz, respectively). Voltage drop across these two segments was measured to obtain their electrical resistance to fluid. Voltage measuring electrodes were placed as follows: for LFV, at the ankle and upper thigh of the right leg, and for TFV, on the midline posterior side of the trunk, at the superior border of the scapula and at the level of the xiphoid process. For both the right leg and thorax, current injecting electrodes were placed one inch away on either side of the voltage measuring electrodes. The fluid volume of a body segment was estimated from its resistance, the distance between the voltage measuring electrodes and the top and bottom circumferences of the segment as described in Yadollahi et al. (2015). The fluid volume of the two legs was obtained by multiplying the fluid volume of right leg by two.

### Measuring lung function

Forced expiratory volume in 1 s (FEV_1_) and forced vital capacity (FVC) were measured in the seated position using a handheld spirometer (Microloop, CareFusion Respiratory Care, CA). The measurements were performed in accordance with ATS/ERS guidelines (Miller et al., 2005), and results were reported using the Global Lung Function Initiative reference values (Quanjer et al., 2012).

### Measuring respiratory impedance

Respiratory system impedance was measured over 5–20 Hz using the impulse oscillometry technique (IOS, CareFusion Respiratory Care, CA). Briefly, small amplitude flow impulses were applied at the subject's mouth during tidal breathing for 30 s, and oscillatory pressure changes were recorded at the mouth to estimate respiratory impedance. Subjects breathed through a mouth piece with their nose clipped and cheeks supported by their palms (Oostveen et al., 2003). Prior to each study measurement, the IOS flow sensor was calibrated using a 3 L syringe.

The IOS parameters used in the analysis were as follows: resistance and reactance at 5 Hz (X5). The detailed explanation of R5 and X5 can be found in Smith et al. (2005) and Bhatawadekar et al. (2017). In short, R5 is a measure of airway obstruction and reflects resistance of the large and small airways, chest wall, and lung tissue. X5 represents the stiffness of the respiratory system and is associated with peripheral airways narrowing (Lutchen et al., 1996).

Measures of sensitivity to fluid shifts were defined as changes in R5 and X5 per unit change in the TFV (ΔR5/ΔTFV and ΔX5/ΔTFV, respectively). The sensitivity values were used to investigate whether for similar amounts of fluid accumulation in the thorax, the changes in R5 and X5 were different among healthy and asthmatic subjects. For comparing sensitivity to fluid shift between men and women, the sensitivity was also obtained as the ratio of changes in R5 and X5 to the changes in the TFV normalized by thoracic surface area, thoracic length and body weight.

### Study design

We designed a randomized, double cross over, day-time protocol to investigate the effects of rostral fluid shifts on lower airway narrowing in asthmatic subjects while supine (Bhatawadekar et al., 2017). Prior to the study measurements, all asthmatic subjects discontinued short-acting β2-agonists for 6 h, long-acting β2 agonists for 24 h, and long acting anticholinergic agents for 72 h as applicable.

During both study arms (control and LBPP), subjects first sat for 30 min, and then lay supine for 30 min. In the supine position, they were randomized to receive LBPP of 40 mmHg from 10 to 30 min, or no pressure (control study arm), and crossed over to the other study arm. While seated, two repeat IOS measurements and spirometry were performed. While supine, two repeat IOS measurements were performed at 0 and 30 min. In both seated and supine positions, the two repeat IOS measurements were averaged to obtain respiratory resistance and reactance. The LFV and TFV were measured continuously. In this analysis, we report the results of the LBPP study arm.

### Statistical analyses

Data are reported as mean ± SD or median [interquartile range]. Normality of data was examined using the Shapiro-Wilk test. If the data was not normal, non-parametric analyses were performed. Differences in baseline data between the healthy and asthmatic subjects were assessed using an unpaired *t*-test or Mann Whitney rank sum test as appropriate. Changes in the IOS measures and fluid volumes in the supine position (0–30 min) within individual groups were assessed using a paired *t*-test or Wilcoxon signed rank test as appropriate. Changes in the IOS measures and fluid volumes between the healthy and asthmatic subjects were compared using two-way ANOVA with time and groups as factors. Differences in baseline IOS measures and fluid volumes between men and women in the two study arms were determined using two-way repeated measures ANOVA with sex and study arm as factors. Changes in R5 and X5 between men and women were compared using two-way ANOVA with time and sex as factors. Correlation between various measures was assessed by Pearson or Spearman correlation analysis as appropriate. *P*-values of < 0.05 were treated statistically significant. All analyses were performed using SAS 5.1 (SAS Institute Inc. Cary, NC).

## Results

In this section, we report new findings of the current analysis. In addition, we present baseline values of fluid volumes and IOS measures and their changes with LBPP as reported in our previous work (Bhatawadekar et al., 2017) for sake of completeness. Compared to the previous study, the new findings include data from a healthy subject and an asthmatic subject (Bhatawadekar et al., 2017).

### Demographics

Eighteen healthy (six women) and sixteen asthmatic (nine women) subjects participated in the study. The two groups showed no differences in the age, height, weight, and BMI (Table 1). Medication details of the asthmatic subjects are reported in Table 2.

**Table 1:** Subject demographics and lung function.

	**Healthy (*n* = 18)**	**Asthma (*n* = 16)**	***p*-value**
Sex (male/female)	**12/6**	**7/9**	
Age, years	51.9 ± 10.5	58.2 ± 9.6	0.08
Height, cm	173.6 ± 10.6	170.1 ± 11.4	0.35
Weight, kg	77.6 [61, 93.5]	80.3 [71.8, 81.6]	0.93
BMI, kg/m^2^	26.0 ± 5.0	27.05 ± 4.1	0.52
**SPIROMETRY (SEATED POSITION)**			
FEV_1_, L	3.55 ± 0.91	2.03 ± 0.60	<0.0001^*^
FEV_1_, % predicted	101.3 ± 12.6	69.0 ± 17.4	<0.0001^*^
FEV_1_ z-score	0.07 ± 0.9	−2.05 ± 1.1	<0.001^*^
FVC, L	4.65 ± 1.0	3.3 ± 0.9	0.0005^*^
FVC, % predicted	105.5 ± 13	85.7 ± 9.9	<0.0001^*^
FVC z-score	0.35 ± 0.9	−0.99 ± 0.7	<0.001^*^
FEV_1_/FVC	76.9 [71.2, 80.2]	68.7 [55.9. 73.03]	0.0011^†^
FEV_1_/FVC % predicted	95.5 [89.0, 100.2]	87.0 [71.3, 90.7]	0.0011^†^
FEV_1_/FVC z-score	−0.4 ± 1.1	−2.03 ± 1.3	0.0008^*^
**FLUID VOLUMES (SUPINE POSITION, 0 MIN)**			
LFV, mL	8857.6 [7206.9, 10125.3]	8883.1 [7946, 9911.9]	0.81
TFV, mL	6271.9 [5490.9, 9773.5]	6351 [4301.3, 9608.6]	0.38
**RESPIRATORY IMPEDANCE (SUPINE POSITION, 0 MIN)**			
R5, cmH_2_O.s.L^−1^	4.4 [3.5, 5.3]	7.5 [6.6, 10.4]	0.0001^†^
X5, cmH_2_O.s.L^−1^	−1.3 [−2.0, −1.0]	−3.3 [−4.6, −2.8]	0.0002^†^

Values are reported as mean ± SD or median [interquartile range] for n subjects. BMI, body mass index; FEV_1_, forced expiratory volume in 1 s; FVC, forced vital capacity, R5 and X5, respiratory resistance and reactance, respectively, at 5 Hz. LFV, leg fluid volume; TFV, thoracic fluid volume. Legs fluid volume indicates fluid volume of both legs as described in Methods. p < 0.05 indicates significant difference between healthy and asthmatic subjects using

* unpaired t-test or

† *Mann-Whitney rank rum test. In the asthma group, leg and thoracic fluid volume is reported for 8 asthmatic women; see results for details*.

**Table 2:** Asthma subjects' medications details.

**Medications**	**No. of subjects**
Short-acting beta-2 agonist (SABA)	1
SABA and Corticosteroid (CS)	2
SABA and a combination of CS and Long-acting β2agonist (LABA)	3
SABA, CS, and a combination of CS and LABA	1
SABA, CS, and a combination of CS and LABA, leukotriene receptor antagonist (LTRA)	1
CS and a combination of CS and LABA	1
A combination of CS and LABA	1
Steroid Nasal Spray, a combination of CS and LABA, LABA and antihistamine	1
Prednisone, SABA, LABA, Thyophyline, Anticholinergic	1
Prednisone, SABA, and a combination of CS and LABA	1
Anticholinergic and a combination of CS and LABA	1
SABA, a combination of CS and LABA, and a monoclonal anti-IgE antibody	1
No medications	1

### Baseline lung function and respiratory impedance

As reported in our previous work (Bhatawadekar et al., 2017), the asthmatic subjects had smaller FEV_1_, FVC, and FEV_1_/FVC than the healthy subjects in the seated position (Table 1). In the asthma group, there was no difference in the absolute and % predicted FEV_1_ between men and women (Table 3). FVC was larger in asthmatic men than in women. However, there was no difference in % predicted values of FVC between the asthmatic men and women (Table 3). Lastly, in the asthma group, both FEV_1_/FVC ratio and FEV_1_/FVC expressed as % of predicted were larger in women than in men.

**Table 3:** Demographics, fluid volumes and lung function in asthmatic men and women.

	**Asthmatic Men (*n* = 7)**	**Asthmatic women (*n* = 9)**	***p*-value**
Age, years	56.9 ± 9.7	59.2 ± 10.0	0.64
Height, cm	177.4 ± 7.8	164.4 ± 11.1	0.02
Weight, kg	85.4 ± 12.8	72.32 ± 10.8	0.11
IBW, % of predicted	1.18 ± 0.14	1.31 ± 0.27	0.27
BMI, kg/m^2^	27.1 ± 3.2	27.0 ± 4.9	0.96
LFV, mL	9708.0 ± 1775.6	8102.6 ± 1661.7	0.09
TFV, mL	8946.7 ± 1955.4	4744.9 ± 1533.3	0.0004
FEV_1_, L	2.1 ± 0.6	1.2 ± 0.6	0.62
FEV_1_, % predicted	59.5 ± 20.8	76.3 ± 10.2	0.05
FVC, L	4.0 ± 0.5	2.7 ± 0.8	0.002
FVC, % predicted	86.8 ± 12.0	84.8 ± 8.7	0.72
FEV_1_/FVC	52.8 ± 12.9	71.1 ± 4.5	0.008
FEV_1_/FVC% predicted	67.4 ± 16.5	89.2 ± 5.2	0.012

*Values are reported as mean ± SD or median [interquartile range] for n subjects. BMI, body mass index; LFV, leg fluid volume; TFV, thoracic fluid volume; FEV_1_, forced expiratory volume in 1s; FVC, force vital capacity; IBW, ideal body weight;. p < 0.05 indicates significant difference between healthy and asthmatic subjects using unpaired t-test. The leg and thoracic fluid volume data is reported for 8 asthmatic women; see results for details*.

In both seated (not shown) and supine position, the magnitude of all IOS measures was significantly larger in the asthmatic subjects than in the healthy subjects at 0 min (Table 1). There were no differences in the baseline R5 (both seated and supine 0 min) between men and women in both healthy and asthma group (*p* > 0.05 for all).

### Sensitivity to fluid shift

**Figure 1:**
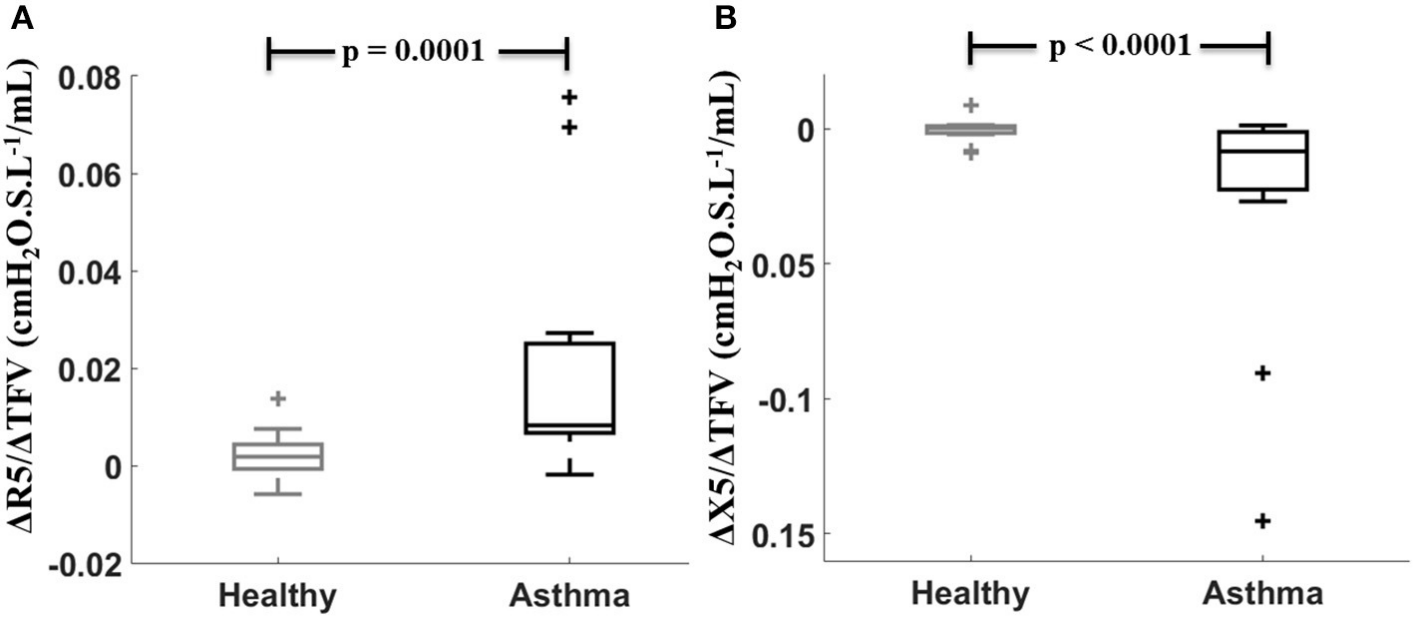
Sensitivity to fluid shift defined by (ΔR5/ΔTFV, **A**) and (ΔX5/ ΔTFV, **B**) compared between healthy (gray) and asthmatic (black) subjects.

Sensitivity values defined by ΔR5/ΔTFV were larger and ΔX5/ΔTFV were smaller (more negative due to X5 being more negative) in the asthmatic subjects than in the healthy subjects (Figure 1). There was no difference in both sensitivity measures between asthmatic men and women (for ΔR5/ΔTFV, *p* = 0.21 and for ΔX5/ΔTFV, *p* = 0.53). Furthermore, the sensitivity measures between the asthmatic men and women were not different when the changes in the TFV were normalized by thoracic surface area (*p* = 0.61 for both), or by the thoracic length (*p* = 0.22 and 0.4), or by body weight (*p* = 0.33 and 0.53, respectively, for R5 and X5 sensitivities).

**Figure 2:**
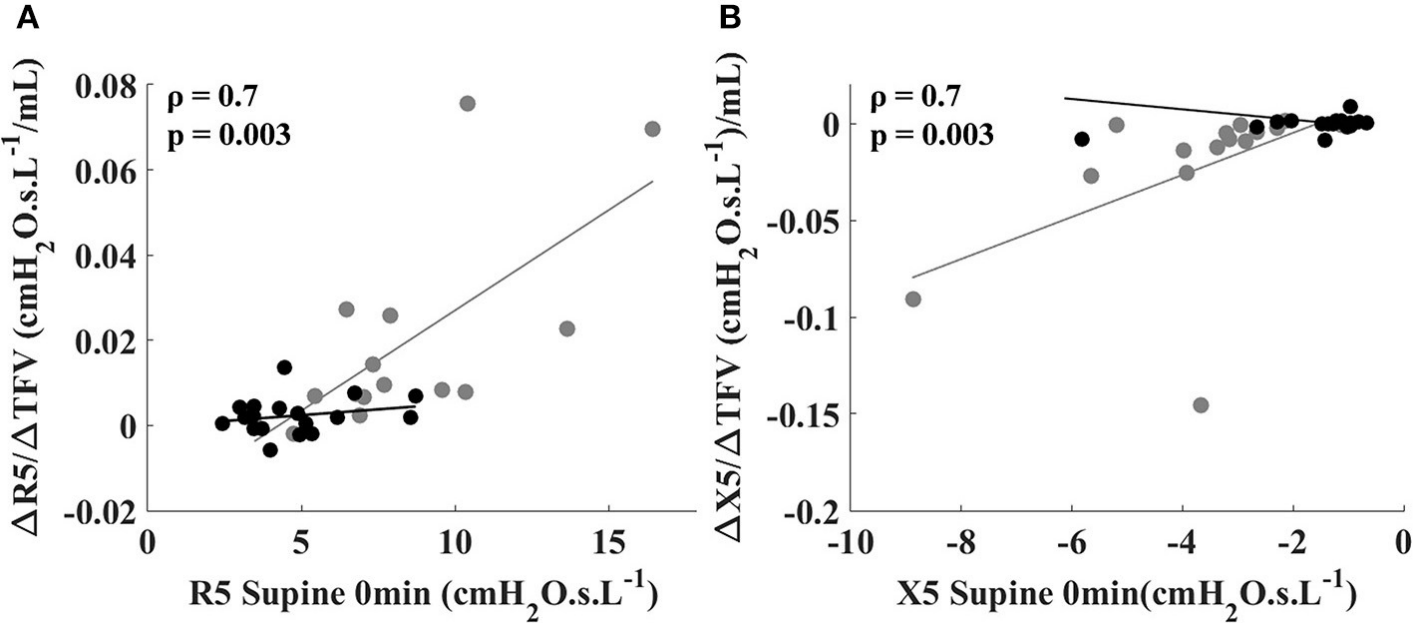
Relationship between sensitivity to fluid shift defined by (ΔR5/ΔTFV) and R5 at 0 min in the supine position **(A)**, and between (ΔX5/ΔTFV) and X5 at 0 min in the supine position **(B)** for healthy (black circles) and asthmatic subjects (gray circles). The lines correspond to the fitted regression equation for each group. Spearman coefficient and *p*-values are shown for asthmatic subjects. For healthy subjects, Spearman coefficient and *p*-values are 0.11 and 0.65 for ΔR5/ΔTFV vs. R5 at 0 min (shown in **A**) and 0.10 and 0.69 for ΔX5/ΔTFV vs. X5 at 0 min (shown in **B**).

In the asthmatic subjects, ΔR5/ΔTFV was significantly correlated with baseline supine R5 (Figure 2A, gray circles). Similar correlation was observed between ΔX5/ΔTFV and baseline supine X5 in the asthmatic subjects (Figure 2B, gray circles). No such correlations were observed between the sensitivity values and baseline supine R5 and X5 in the healthy subjects (Figures 2A,B black circles).

### Baseline fluid volumes and changes in fluid volumes with LBPP

Baseline LFV and TFV (supine 0 min) were similar between the healthy (*n* = 18) and asthmatic subjects (*n* = 15, LFV data from one asthmatic subject and TFV data from another asthmatic subject were not available; Table 1). With LBPP, LFV decreased, and TFV increased in both groups (Table 4).

**Table 4:** Changes in fluid volumes and IOS measures with lower body positive pressure (LBPP) in healthy and asthmatic subjects.

	**Healthy (*n* = 18)**	**Asthma (*n* = 16)**	***p*-value time × group**
ΔLFV, mL	364.9 [313.9, 422.5]^#^	334.6 [220.8, 485.9]^#^	0.26
ΔTFV, mL	170.2 ± 79.9^#^	118.0 ± 88.53^#^	0.11
ΔR5, cmH_2_O.s.L^−1^	0.4 ± 0.6^#^	1.3 ± 0.9^#^	0.0012
ΔX5, cmH_2_O.s.L^−1^	0.05 [−0.2, 0.1]	−0.7 [−1.1, −0.2]^#^	0.0005

Values are reported as mean ± SD or median [interquartile range]. Δ, changes with LBPP, LFV, legs fluid volume; TFV, thoracic fluid volume; R5 and X5, respiratory resistance and reactance, respectively, at 5 Hz. p < 0.05 indicates significant interaction effect between healthy and asthmatic subjects using two-way ANOVA, and

# *represents significant change (p < 0.05) following lower body positive pressure (LBPP) within each group*.

There was no difference in the baseline LFV between the asthmatic men (*n* = 7) and women (*n* = 8) in the LBPP study arm. However, the baseline TFV was larger in the asthmatic men (*n* = 7) than in the women (*n*=8) (Table 3). With LBPP, in both asthmatic men and women, the LFV reduced (ΔLFV, 372.5 ± 114.2 mL, *p* = 0.0001 and 333.1 ± 143.5 mL, *p* = 0.0003), and TFV increased (ΔTFV, 156.2 ± 110.4 mL, *p* = 0.01 and 85.4 ± 50.6 mL, *p* = 0.002). The changes in the leg and TFV between the asthmatic men and women were not different (LFV: *p* = 0.45, TFV: *p* = 0.1).

### Changes in respiratory impedance with LBPP

The changes in IOS measures R5 and X5 have been reported in our previous work (Bhatawadekar et al., 2017), and reproduced with the inclusion of data from recently recorded healthy and asthmatic subject in Table 1.

**Figure 3:**
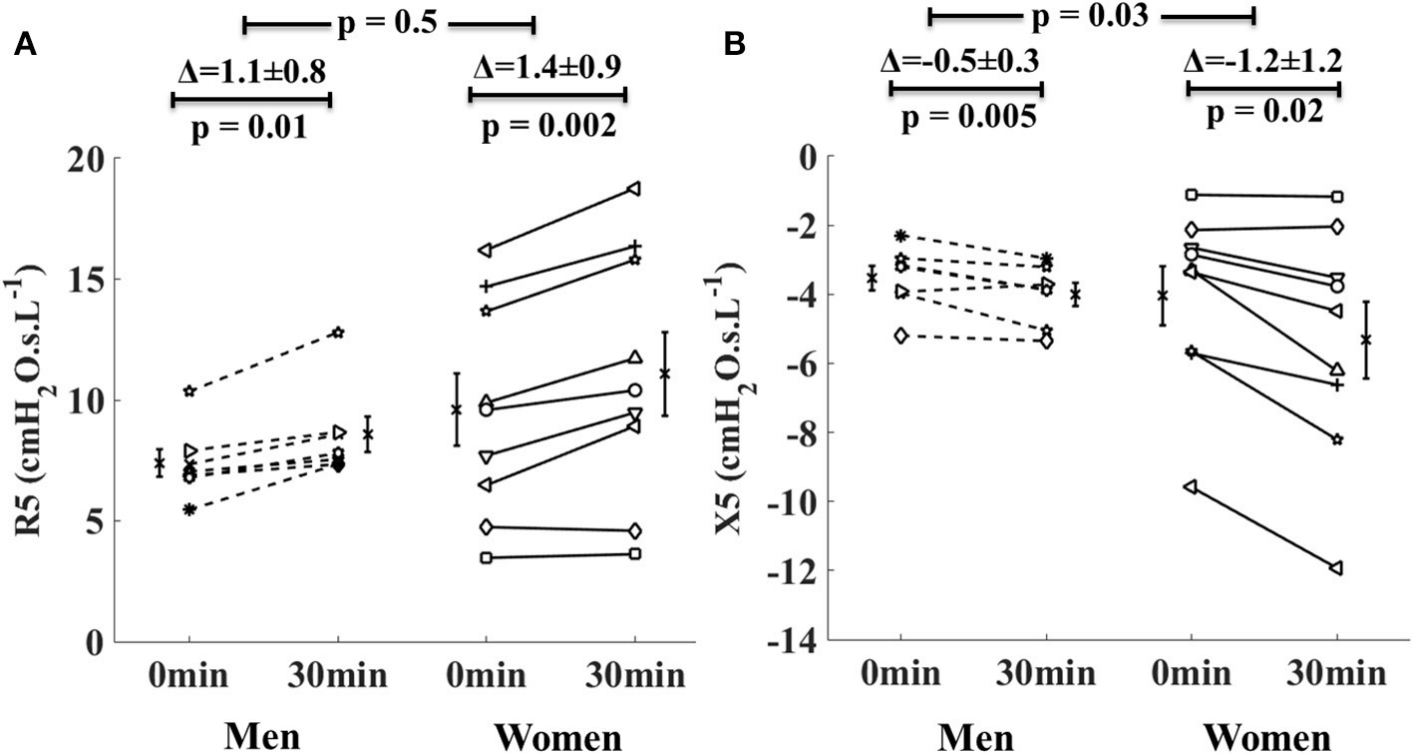
Changes in the respiratory system resistance at 5 Hz (R5, **A**) and reactance at 5 Hz (X5, **B**) in men with asthma (dotted lines) and women with asthma (solid lines) from 0 to 30 min during lower body positive pressure (LBPP) study arm. Each line represents an individual subject. The bars on either side of the data lines represent group mean value with standard error of mean.

The new finding here is that in the asthma group, R5 increased and X5 decreased (became more negative) in both men and women. While the increases in R5 were similar between men and women, the decreases in X5 in women were two times greater than in men (Figure 3). Furthermore, comparing the airway mechanics between obese women (*n* = 3, BMI = 32.1 ± 0.8 kg/m^2^, mean ± SD) and non-obese women (*n* = 5, BMI = 24.8 ± 3.7 kg/m^2^) in the asthma group, we found that baseline R5 was significantly larger (13.5 cmH_2_O.s.L^−1^ vs. 7.6 cmH_2_O.s.L^−1^, *p* = 0.03) and baseline X5 was borderline more negative (−6 cmH_2_O.s.L^−1^ vs. 2.8 cmH_2_O.s.L^−1^, *p* = 0.06) in the obese women than in the non-obese women. In the obese asthmatic women, there was a trend for larger R5 at 30 min following intervention (15.7 cmH_2_O.s.L^−1^ vs. 8.7 cmH_2_O.s.L^−1^, *p* = 0.07) and more negative X5 (−7.4 cmH_2_O.s.L^−1^ vs. −3.9 cmH_2_O.s.L^−1^, *p* = 0.18) compared to the non-obese asthmatic women (Figures not shown here).

## Discussion

The main findings of this retrospective analysis are as follows: in response to application of LBPP, (1) In the asthmatics, smaller baseline airway caliber, as assessed by larger R5, was associated with increased sensitivity to fluid shift in the supine position; and (2) the asthmatic women showed larger reduction in X5 than asthmatic men.

Our previously published work suggests a new mechanism that can contribute to nocturnal exacerbations of asthma by demonstrating the mechanistic link between rostral fluid shifts and lower airway narrowing in asthma patients while supine (Bhatawadekar et al., 2017). However, it does not specifically answer why similar airway narrowing does not happen in healthy subjects, or why all asthmatics do not experience airway narrowing due to rostral fluid shift. The current complements our previous work (Bhatawadekar et al., 2017) by investigating the risk factors for the adverse effects of fluid accumulation in the thorax on lower airway narrowing and which patient populations may be at higher risk.

Our findings suggest that baseline airway caliber in the supine posture is an important predictor of the amount of lower airway narrowing induced by fluid shifts from the lower body to the thorax. They imply that patients with narrow airways at baseline such as asthmatic women, obese asthmatics, or patients with severe asthma may be more sensitive to the effects of overnight fluid shifts during sleep. The results of this study, when confirmed in overnight studies, will help us design targeted therapies to reduce fluid retention in the legs and nocturnal rostral fluid shift in high risk patient populations preventing the adverse effects of fluid shifts.

Previous studies suggest that geometric factors contribute to the airway hyperresponsiveness in asthma (Benson, 1975). Airway hyperresponsiveness to methacholine (Ichinose et al., 2000) and histamine (Cartier et al., 1982; Grimm et al., 2000) correlates positively with baseline airway caliber, and improves following an increase in the airway caliber (Ichinose et al., 2000). Indeed reduced caliber of the intrathoracic airways is an independent predictor of airway reactivity in asthma (Sears et al., 2003) and in the general population (Britton et al., 1994). Our results suggest that in asthmatic subjects, airway caliber in the supine position predicts the airway response to rostral fluid shift. Thus, asthmatic subjects with narrower airways at baseline (higher baseline respiratory resistance) such as those with severe asthma may experience greater airway narrowing with fluid shift to the thorax compared to those with normal airway caliber.

The strong positive association between baseline airway caliber and sensitivity to fluid shift observed in the asthmatic subjects can be explained by the inverse relationship between airway radius and airway resistance (According to Poiseuille equation, resistance varies inversely with the fourth power of the radius; Kaminsky, 2012). Accordingly, a similar amount of fluid accumulation in the airway wall may cause a larger reduction in the caliber of small airways than in the large airways, and consequently result in a greater increase in the airway resistance of individuals with a narrower airway tree.

In contrast to the asthmatic subjects, there was no correlation between the baseline airway caliber and sensitivity to fluid shift in the healthy subjects. This may be due to larger baseline airway caliber in the healthy subjects as opposed to the asthmatic subjects. Of note, there can be big differences in the airway caliber based on age, sex, and height in a normal population (Quanjer et al., 2012). Thus, airway caliber may not be the only explanation for our results. The difference in airway response to fluid shift between healthy and asthmatic subjects may also relate to other features of asthma that are not explained. In any case, the effect of increased thoracic fluid volume on airway resistance was trivial in the healthy subjects compared to the asthmatic subjects.

In the asthmatic subjects following LBPP, women experienced larger increase in the stiffness of the respiratory system than men (indicated by two time greater reduction in X5 in the asthmatic women than in the asthmatic men, Figure 3). Indeed, it is known that for the same lung size and thoracic gas volume, women have smaller airway luminal areas, and thus slightly higher respiratory resistance values than men. This may suggest that in asthma, greater reduction in X5 in women was associated with baseline small airway narrowing. Similarly, among the asthmatic subjects, obese women had narrower baseline airway caliber than non-obese women (assessed by R5). There was a trend for higher airway response to fluid shift in the obese asthmatic women compared to the non-obese asthmatic women, but it did not reach statistical significance due to the small sample size. These results are interesting and further evaluation is needed in a larger group of asthmatic men and women including obese women with asthma.

Our work has raised important research questions leading to impetus for future work. The airway response and sensitivity to fluid shift in asthma observed in our study may also be associated with vagally-mediated reflex bronchoconstriction. Future studies should investigate this question.

Our findings suggest that asthmatic subjects sensitive to the effects of rostral fluid shift during sleep may particularly benefit from the treatments aimed at reducing daytime leg fluid accumulation such as walking exercise (Mendelson et al., 2016), by wearing compression stockings (White et al., 2015), or using diuretics during the day (Kasai et al., 2014). Future studies should examine whether in asthma, prevention of leg fluid retention during the day and subsequent reduction in overnight fluid shift to the thorax improves nocturnal airway narrowing in asthma and if asthmatic women respond more to the intervention than asthmatic men.

A limitation of this study is that we did not measure lower airways caliber, but used respiratory resistance measured by IOS as a surrogate measure of lower airways caliber (Tattersfield and Keeping, 1979; Smith et al., 2005). An advantage of using IOS is that it is minimally invasive, and allows repeated assessment of lung mechanics in a wide range of patient population. Other commonly used measures of airway caliber include FEV_1_, peak expiratory flow rate, and forced expiratory flow at 25–75% of FVC measured during spirometry (Lehane, 1982). However, deep inspirations required during spirometry may change airway caliber (Nadel and Tierney, 1961). Some studies have used high resolution computed tomography (HRCT) to measure changes in the airway caliber *in vivo* in animals (Brown et al., 1995a,b) and humans (Okazawa et al., 1996; Brown et al., 2000), and resolved airways as small as 1–2 mm in diameter. While HRCT can measure changes in the airway caliber that are not detectable by spirometry or IOS, it involves radiation and thus may not be ethical in human research studies such as this.

In conclusion, we have shown that in asthma, sensitivity to rostral fluid shift is associated with the baseline airway caliber in the supine position, and small caliber airways narrow more than the large caliber airways in response to fluid shift to the thorax. Secondly, we have shown that asthmatic women and obese asthma patients may constitute a population of increased risk for small airway narrowing with rostral fluid shift. Our study can help phenotype the patients into subgroups that may be at the increased risk for the adverse effects of fluid shifts and benefit from the treatments designed to reduce fluid accumulation in the legs.

## Author contributions

SB and AY performed experiments. SB and GK analyzed data. SB, GK, CF, MI, JF, ST, MS, OL, and AY interpreted results of experiments. SB prepared figures. SB drafted manuscript. SB, GK, MI, JF, ST, and AY edited and revised manuscript. SB, GK, CF, MI, JF, ST, MS, OL, and AY approved final version of manuscript. AY conceived and designed research.

### Conflict of interest statement

AY, MI, and ST received a grant from by Ontario Lung Association, Canadian Respiratory Research Network, CIHR Training Grant in Sleep and Biological Rhythms and AllerGen. GK was supported by a scholarship from Science without Borders/CNPq-Brazil. The other authors declare that the research was conducted in the absence of any commercial or financial relationships that could be construed as a potential conflict of interest.
